# Treatment of deep caries lesions: a multi-center randomised controlled trial using a comprehensive set of outcome parameters

**DOI:** 10.1186/1745-6215-16-S1-P6

**Published:** 2015-05-29

**Authors:** Falk Schwendicke, Sebastian Paris, Hardy Schweigel

**Affiliations:** 1 Department of Operative and Preventive Dentistry, Charité–Universitätsmedizin, Berlin, 14197 Berlin, Germany; 2 DMG Dental Material Gesellschaft, Department of Clinical Research, 22547 Hamburg, Germany

## Background

For treating deep caries lesions, selective or stepwise excavations seem advantageous compared with complete caries removal, but current evidence is sparse and focusses mainly on clinical success. Given that no core outcomes are defined for trials investigating caries removal and restorative dental treatments, we aim to compare different excavations of primary teeth using clinical, patient-centered and economic outcomes.

## Methods

We plan a prospective multi-center, two-arm parallel group, randomised controlled trial comparing selective and stepwise excavation in sensible, asymptomatic deciduous molars with deep caries lesions. Trial outcomes were decided after systematic assessment of the literature and discussions with clinicians, patients and health economists.

## Results

We will recruit 300 children aged 3-9 with minimum one deeply carious molar. After inclusion, sequence generation will be performed. During initial treatment, leathery, moist and reasonably soft dentin will be left in proximity to the pulp followed by adhesive restoration. Afterwards, patients’, dentists’ and parents’ assessment of the treatment will be recorded using visual-analogue- and Likert-scales, respectively. Treatment and opportunity costs will be calculated. Re-examination will be performed after six months. Then, teeth will be allocated to one of the two interventions. Selectively excavated teeth will not be treated further, whilst for stepwise caries removal, a second excavation will be performed until only hard dentin remains. Clinical re-evaluation will be performed after 12, 24 and 36 months. Restorations will be re-assessed using modified Ryge-criteria. Objectively or subjectively required re-treatments will determine success or survival. Re-treatments will be evaluated both subjectively and regarding the generated costs.

## Conclusions

Available core outcomes for caries trials are not applicable for comparing different excavations. The planned trial will comprehensively assess different treatments for deep lesions, and could identify discrepancies between conventionally used and alternative outcome parameters.

## Trial registration

Clinicaltrials.gov *NCT02232828*

